# Finding malaria hot-spots in northern Angola: the role of individual, household and environmental factors within a meso-endemic area

**DOI:** 10.1186/1475-2875-11-385

**Published:** 2012-11-22

**Authors:** Ricardo J Soares Magalhães, Antonio Langa, José Carlos Sousa-Figueiredo, Archie CA Clements, Susana Vaz Nery

**Affiliations:** 1 Infectious Disease Epidemiology Unit, School of Population Health, University of Queensland, Herston, Queensland, Australia; 2 Centro de Investigação em Saúde em Angola, Caxito, Rua Direita do Caxito, Hospital Provincial do Bengo, Caxito, Angola; 3 Disease Control Strategy Group, Liverpool School of Tropical Medicine, Liverpool, UK; 4 Department of Infectious and Tropical Diseases, London School of Hygiene & Tropical Medicine, London, UK

**Keywords:** Risk mapping, Malaria, Socio-economic factors, Physical environment, Disease control

## Abstract

**Background:**

Identifying and targeting hyper-endemic communities within meso-endemic areas constitutes an important challenge in malaria control in endemic countries such like Angola. Recent national and global predictive maps of malaria allow the identification and quantification of the population at risk of malaria infection in Angola, but their small-scale accuracy is surrounded by large uncertainties. To observe the need to develop higher resolution malaria endemicity maps a predictive risk map of malaria infection for the municipality of Dande (a malaria endemic area in Northern Angola) was developed and compared to existing national and global maps, the role of individual, household and environmental risk factors for malaria endemicity was quantified and the spatial variation in the number of children at-risk of malaria was estimated.

**Methods:**

Bayesian geostatistical models were developed to predict small-scale spatial variation using data collected during a parasitological survey conducted from May to August 2010. Maps of the posterior distributions of predicted prevalence were constructed in a geographical information system.

**Results:**

Malaria infection was significantly associated with maternal malaria awareness, households with canvas roofing, distance to health care centre and distance to rivers. The predictive map showed remarkable spatial heterogeneity in malaria risk across the Dande municipality in contrast to previous national and global spatial risk models; large high-risk areas of malaria infection (prevalence >50%) were found in the northern and most eastern areas of the municipality, in line with the observed prevalence.

**Conclusions:**

There is remarkable spatial heterogeneity of malaria burden which previous national and global spatial modelling studies failed to identify suggesting that the identification of malaria hot-spots within seemingly mesoendemic areas may require the generation of high resolution malaria maps. Individual, household and hydrological factors play an important role in the small-scale geographical variation of malaria risk in northern Angola. The results presented in this study can be used by provincial malaria control programme managers to help target the delivery of malaria control resources to priority areas in the Dande municipality.

## Background

Boosted by increased international funding and greater political commitment, several countries in Africa with high-burden malaria have achieved in recent years high coverage of their at-risk populations with effective mosquito control interventions and access to artemisin-based combination therapy (ACT) [1]. Despite these efforts morbidity and mortality caused by malaria remains high in 67 of the 99 countries in which malaria is endemic [2].

In highly endemic countries, such as Angola, the current main focus of interventions is to reduce the disease burden to a level at which it is no longer a public health problem. Angola is estimated to have 3.4 million cases of malaria annually, mainly caused by *Plasmodium falciparum*[3,4]. Malaria transmission in Angola occurs all year round, but is highest in northern provinces while the southern provinces have highly seasonal or epidemic malaria [4]. Here, malaria is thought to be responsible for 35% of mortality in children under the age of five, 25% of maternal mortality, and 60% of hospital admissions for children under five [4,5].

National guidelines currently in place for malaria control include indoor residual spraying (IRS) in selected urban districts (covering over 100,000 households and 4% of the population at risk); free distribution of long-lasting insecticidal nets (LLINs) at neonatal consultations, and free ACT at public health facilities, with over four million doses delivered in 2007–08, enough to treat almost 70% of the reported cases [3]. These efforts in the last few years have led to a reduction of about 60 percent in the number of deaths caused by the disease in the country [4]. Furthermore, based on the results of national malaria indicator surveys, between 2006 and 2011 the prevalence of *P*. *falciparum* infection in children under five declined from 21% to 13% [4].

Despite the progress achieved, there are still major challenges related to the control and eventual elimination of malaria, particularly in endemic foci. Recently, a sub-national malaria survey was conducted in a meso-endemic area in Northern Angola (Dande municipality, Bengo province) [6]. The results of this study revealed that children were at a significantly increased risk of *Plasmodium* spp. infections (18.3% versus 9.3%) and parasitaemia (309.0 versus 194.4 parasites/μL) compared to female adults. It also revealed significant differences in endemicity between the communes (the lowest administrative level) included in the analysis.

The existence of unidentified hyper-endemic communities within meso-endemic areas constitutes an important challenge in the control or elimination of malaria [7]. Modern spatial analysis methods provide tools for the identification and quantification of the population at risk of parasite infections in endemic communities [8,9]. In the case of malaria, these tools have been used to model and develop malaria infection maps at different spatial scales [10-24]. These include continental and national spatial models of malaria infection constructed using data from a sample of the population with interpolation to areas where infection data are absent. At the highest scale, researchers have used a global model of malaria and spatially predicted malaria parasite rates for children aged 2–10 years [10,25]. More recently, malaria indicator survey (MIS) data from Angola for 2007 were used to produce a national level predictive risk map for the country [26]. However, large uncertainties surround these efforts particularly at small-spatial scales at which most malaria control programmes operate.

A recent mathematical modelling study has shown that targeting hotspots within areas of lower endemicity with LLINs and IRS could help achieve malaria elimination, while untargeted interventions with the same resources would lead to more modest reductions in malaria parasite prevalence [7]. Therefore, for current global and national malaria models to be useful spatial decision support tools for targeted malaria surveillance and intervention delivery, they need to provide sufficient detail (i.e. at the appropriate decision-making spatial resolution) to identify areas where malaria endemicity is at its highest. One way of determining the need for higher resolution malaria mapping is to empirically evaluate the performance of larger-scale models to that of local, sub-national models at identifying communities that require targeted malaria interventions.

The study aimed to quantify the role of individual-level factors and physical environment in the small-scale geographical variation of malaria, generate a predictive prevalence map for children aged <15 years and compare this map with previous continental and national mapping efforts to observe the need for local spatial prediction studies. It also aimed to provide a high-resolution cartographic resource that can help identify malaria hot-spots to enable the national malaria control programme to plan and implement targeted malaria interventions within each commune of Dande municipality, Angola.

## Methods

### Ethics statement

The study protocol was approved by the Angolan Ministry of Health Ethics Committee.

One day before the survey, a field worker visited each household to provide explanations on the study and obtain individual informed consent, signed or marked with a fingerprint from the selected mother or caregiver. All households that agreed to participate were enrolled in the study after handing in the signed consent form. Participants found to be positive for *P. falciparum* infection were treated with ACT.

### Population general information

The Demographic Surveillance System (DSS) established in 2009 within the CISA project (*Centre for Health Research in Angola*, translated) in the Dande municipality, aims to collect longitudinal data on the population’s structure, dynamics and geographical location [6,27]. Dande municipality, Bengo Province, north-western Angola, is a largely rural area 60 km north of the capital Luanda. According to the census conducted between September 2009 and April 2010 this area of about 4,700 km^2^, has 59,844 registered inhabitants in 15,604 households distributed in 69 hamlets. The study area of the CISA DSS includes three of five communes (Caxito, Mabubas and Úcua) within Dande municipality.

### Survey methods

A community-based cross-sectional epidemiological survey in the CISA DSS area was conducted between May and August 2010 [6]. Detailed information about the sampling design and survey management are detailed in Sousa-Figueiredo *et al.*[6]. Briefly, a total of 972 households were selected and included in the study, distributed in 36 hamlets, including a total of 960 mothers (mean age 33.3 years, range 16 to 80 years) and 2,379 children aged ≤15 years (mean age 5.9 years, range 6 months to 15 years) in Caxito (794 children), Mabubas (904 children), and Úcua (681 children) [6]. Prior to the survey, field workers were trained to interview caregivers using a questionnaire, which collected information relating to the caregiver and her children. Questions included: demographic information (age, sex), occupation, access to healthcare and history of previous treatment, and questions related to malaria (e.g. knowledge and bed-net ownership and utilization). Malaria parasitaemia was detected by preparing blood smears in the field which were stained with 10% Giemsa. Malaria parasites were screened by two independent microscope technicians using a double-blind approach [28]. In the field participants were tested using a rapid diagnostic test – *Paracheck-Pf* (Orchid, India) allowing for treatment of positive cases.

### Geolocation of households

**Figure 1:**
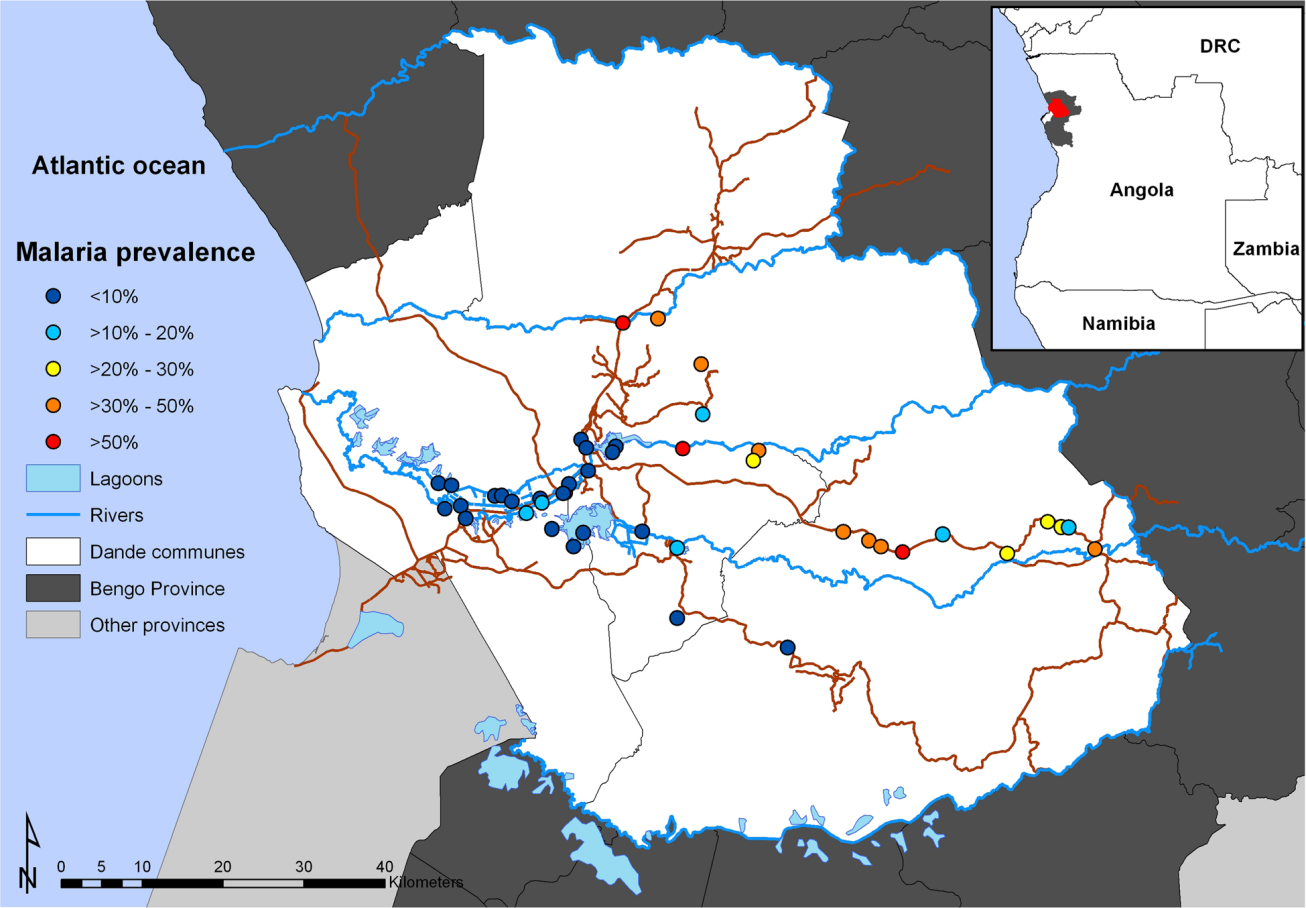
Geographical distribution of malaria in children aged <15 years in the Dande municipality in Angola.

The geographic information available in the DSS data warehouse included the coordinates (longitude and latitude) of the majority of the households which were extracted on-site using Garmin® GPSMAP® 60Cx handheld global positioning system (GPS) receivers, and household coordinates were collected with a precision of two to six metres. The codes of houses included in the survey were linked to the CISA DSS data warehouse to obtain household information, which included type of flooring and ceiling. Because 30% of households included in the survey did not have geographic coordinates the geographical centre of the sector (the lower subdivision of the hamlet) was used as the geographical unit for the analysis. Centroids were estimated based on a digitalized map of the hamlets, using the spatial analyst tool in the GIS. Due to their large size, six hamlets are divided into sectors (subdivision of the hamlet) and in these cases the centroid of the sector was used as the geographical location for the analysis. This enabled 95% of individuals included in the survey to be successfully georeferenced to a total of 43 locations. Figure 1 shows the geographical location of the centroids of the 43 locations where the prevalence survey was conducted.

### Data on the physical environment

A shapefile of large perennial inland water bodies was obtained from the Food and Agriculture Organization of the United Nations [29]. The shapefile of large perennial inland water bodies was updated by adding the location of rivers, lagoons and irrigation canals in the study area. The location of rivers, lagoons and irrigation canals was obtained by recording the path of these features on the GPS with 10-metre intervals. Layers for these features were created by uploading the GPS information on the geographical information system (GIS) ArcGIS 9.3.1 (ESRI, Redlands, CA) and using the vectorization procedure in the GIS. The distance to rivers, lagoons, large perennial water bodies and irrigation canals was extracted for each sector in the GIS ArcGIS version 10.0 (ESRI, Redlands, CA). Electronic data for land surface temperature and rainfall for a 1 km × 1 km grid cell resolution were also obtained from the WorldClim data warehouse. A 5km resolution population surface derived from the Global Rural–urban Mapping Project (GRUMP) beta product was obtained from the Center for International Earth Science Information Network (CIESIN) of the Earth Institute at Columbia University [30]. The geographical centre of the sector was linked to the environmental data to obtain values of the environmental variables for each sector using the spatial overlay procedure in the GIS.

### Variable selection and residual spatial variation

For the purpose of the analysis the presence of malaria parasites in blood identified by the Giemsa method was used as the outcome variable of the analyses and thus all children were categorized into infected and not infected based on the presence of at least one parasite. In order to explain the spatial variation in malaria risk, the individual level variables age, sex, maternal knowledge of malaria, bed net usage and previous malaria treatment and five household variables: type of household walls, type of household roof, number of rooms in household, type of water source and type of sanitation facility were considered in the initial variable screening stage. These household variables were considered important as they could represent proxies for the economic status of the family as well as a measure of household exposure to malaria vectors. Environmental data such as land surface temperature, rainfall, distance to rivers, distance to irrigation canals, distance to lagoons and distance to health care centers were also considered in the variable screening stage.

Univariate logistic regression models for a Bernoulli-distributed outcome and cluster correction by hamlet using robust standard errors were built to screen variables for inclusion in the final multivariable model, using the statistical software Stata version 10.1 (Stata corporation, College Station, TX). During this stage, variables with *P*>0.2 were excluded from further analysis; using that criterion, bed net usage, previous malaria treatment, the household variables type of household walls, number of rooms in household and type of water supply, and the variable distance to irrigation canals were subsequently excluded.

Spatial dependence in the residuals of the final non-spatial multivariable model was investigated using a residual semivariogram which is a graphical representation of the spatial variation left unexplained by the covariates included in the model. The residuals of the final non-spatial multivariable model were extracted and analysed for spatial dependence in the statistical software R using the geoR package version 2.14.1 (The R foundation for statistical computing).

### Spatial risk prediction and model validation

A Bayesian geostatistical model of malaria prevalence was developed in WinBUGS 1.4 (Medical Research Council, Cambridge, UK and Imperial College London, UK) (see Additional file 1 for detailed model specification). The model included an intercept, the individual level variables age, sex and maternal knowledge of malaria, the household variables type of household roof and type of sanitation facility, the environmental variables land surface temperature, rainfall, distance to rivers, distance to lagoons and distance to health care centers, and a geostatistical random effect. The covariate effects were summarized by using the mean and 95% credible intervals (representing the range of values that contains the true value with a probability of 95%). The geostatistical random effect modelled spatial correlation as a function of the separating distance between pairs of hamlets. Model predictions were used to generate a malaria risk map for boys of older ages (the subgroup with the highest risk of malaria) for the entire study area in ArcGIS version 10.0. The prediction model included the individual level variables age and sex and the variables of the physical environment temperature, rainfall, distance to lagoons, distance to health care centre and distance to rivers. While using age and sex allowed predicting to the subgroup most at risk to malaria in the study area (i.e. boys or older age), the use of the variables of the physical environment allowed prediction across a continuous landscape. To determine the discriminatory performance of the model predictions relative to observed prevalence thresholds (10% and 50%), the area under the curve (AUC) of the receiver operating characteristic was used. An AUC value of >0.7 was taken to indicate acceptable predictive performance [31]. A map of predicted standard deviation was also generated to depict the uncertainty around the mean predicted malaria endemicity.

### Comparison with previous larger-scale studies in the region

The global malaria map for 2011 [10] was included in the GIS and the shapefile of the administrative boundaries of the Dande municipality was used as a mask to extract the spatial distribution of malaria for the study area (see Additional file 1). The mean malaria endemicity in the malaria risk map and in the malaria map for the study area from the global malaria map for 2011, was estimated based on the summary statistics provided by the raster properties in the GIS. The national malaria map for Angola using MIS from 2007 [26] was not available to us and comparisons between this map and malaria risk map developed in this study were made visually. The comparison between spatial distributions of predicted malaria endemicity for the different studies was made visually.

### Calculation of numbers of children at-risk of malaria for 2011

To estimate the number of children at-risk of malaria in the six communes of Dande municipality for 2011, complete and up-to-date numbers of children aged ≤15 years was retrieved from the CISA DSS database. However this information was not available for the communes of Barra do Dande and Quicabo; for these communes the predictive malaria risk map was used and multiplied it by a population map of ≤15 yrs for 2011. This population map was generated by multiplying a population map for 2009 (see methods for details) by the population growth factor for Angola for the period 2010–2015 and by the average proportion of ≤15yrs in Angola for the period 2010–2015, available from the UN World Population Prospects website [32].

## Results

### Data for analysis

A total of 2,265 children (95.5%) aged ≤15 years were geolocated to 43 locations in the study area (Figure 1). The mean observed prevalence of *Plasmodium* parasitaemia in the study area was 18.3%. The mean age in years was 6.5 (SD: 3.8) (Table 1). Eight-two percent of mothers reported being aware of malaria, 47% reported having previously being treated or treated their children for malaria, and 28% reported owning bed-nets. Most children lived in households in which the main source of water supply was the river (54%). Forty-eight percent lived in households without access to latrines, 53% in households with walls made of adobe and 69% in households with roof made of metal sheets.

**Table 1:** Characteristics of 2,265 children aged ≤15 years included in the analysis

**Variables**	**Total**	**Malaria**	
		**No**	**Yes**
**Age**			
mean		6.5	6.4
min-max		0.6 - 15	0.9-15
**Gender**			
Male	1,094	880	214
Female	1,171	978	193
**Maternal malaria awareness**			
No	394	297	97
Yes	1,843	1,540	303
**Previous malaria treatment**			
No	1,134	908	226
Yes	1,024	876	148
**Bednet ownership**			
No	1,621	1,307	314
Yes	617	531	86
**Water source**			
Public tap	158	156	2
Private tap	40	40	0
River	1,223	1,024	199
Irrigation canal	5	5	0
Lagoon	134	131	3
Cacimbo	525	342	183
Well	41	32	9
Other	139	128	11
**Sanitation**			
No latrine	1,078	904	174
Latrine with water supply	397	381	16
Latrine without water supply	790	573	217
**Type of household walls**			
Adobe	1,209	1,027	182
Bricks/blocks	277	262	15
Wattle and daub	454	304	150
Wattle	207	151	56
Wood	1	1	0
Other (metal sheet or plastic)	117	113	4
**Type of household roof**			
Straw	452	314	138
Metal sheet	1,565	1,306	259
Tiles	66	65	1
Other (canvas or plastic)	182	173	9
**Number rooms in household**			
mean		2.5	2.2
min-max		1-8	1-6

### Spatial risk prediction

**Figure 2:**
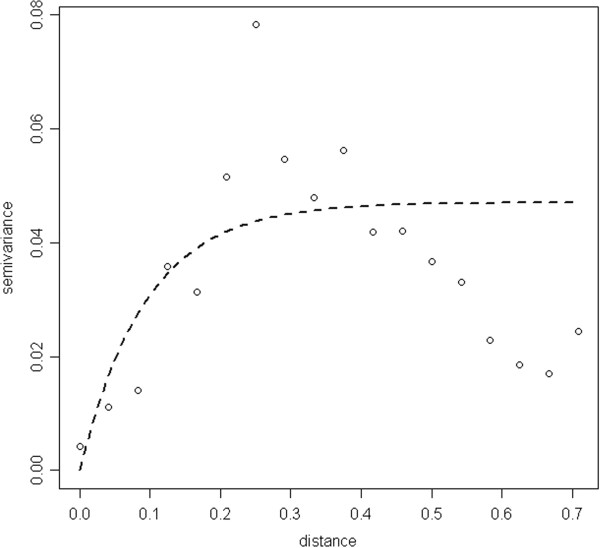
Residual semivariogram of final multivariable model (non-spatial) of malaria prevalence in children aged <15 years in the Dande municipality, Angola.

**Figure 3:**
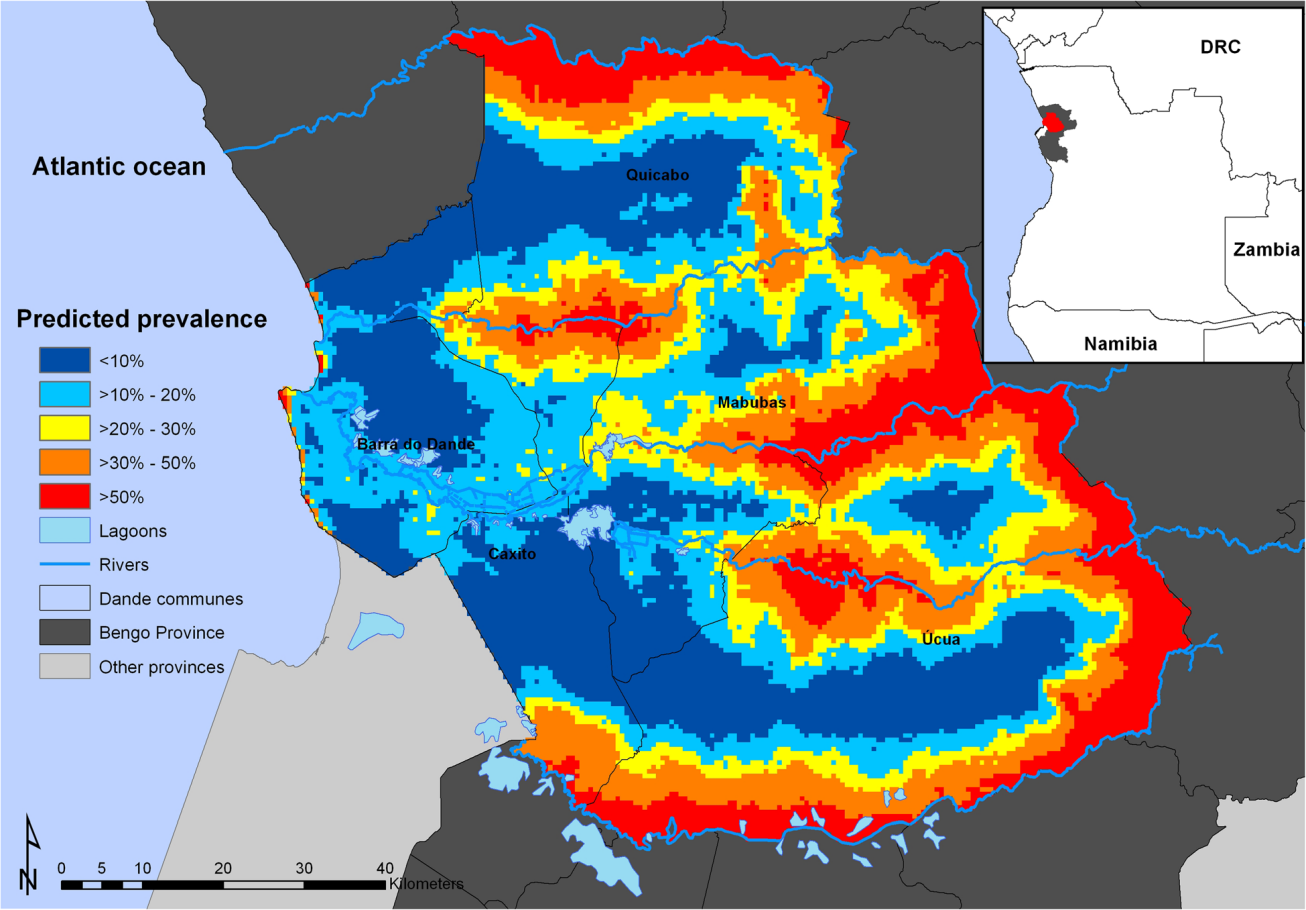
Predicted spatial distribution of malaria in boys of older ages for the Dande municipality in Angola.

The residual semivariogram of the final (non-spatial) multivariable model revealed significant small scale spatial variation still unaccounted for by the variables in the final multivariable model in that the practical range was 0.281 and the sill was 0.047 (Figure 2). For the purpose of spatial modelling 2,158 children aged ≤15 years with complete information regarding community geolocation (i.e. sector coordinates), demography (i.e. age and gender), information about maternal malaria awareness and previous treatment for malaria and household information (i.e. type of wall and roof of household, number of rooms in the household, and type of water supply and sanitation) were included. Results of the Bayesian geostatistical model of malaria prevalence indicated that (Table 2): prevalence of *Plasmodium* infection was positively associated with age, household with access to latrines without water, households with straw roofing, rainfall, distance to lagoons and health care centres, and was negatively associated with being female, maternal malaria awareness, households with access to latrines with water, households made of adobe, households with canvas roofing, temperature, and distance to rivers. These associations were only significant for maternal malaria awareness, households with canvas roofing, distance to health care centre and distance to rivers. Results also indicate that the rate of decay of spatial autocorrelation [Phi (*φ*)] was 12.68. This means that, after accounting for the effect of covariates, the radii of the clusters were approximately 26km (note, *φ* is measured in decimal degrees and 3/*φ* determines the cluster size; one decimal degree is approximately 111 km at the equator). Large high-risk areas of malaria infection (prevalence >50%) were found in a region covering the north and most of the eastern areas of the Dande municipality (Figure 3).

**Table 2:** Spatial effects for prevalence of malaria in children aged ≤15 years in Dande municipality, Angola

**Variable**	**Mean (95% CrI)**
**Age in years**	0.03 (−0.02, 0.07)
**Female** (vs Male)	−0.17 (−0.47, 0.15)
**Maternal malaria awareness** (vs not aware)	−0.44 (−0.85, -0.02)
**Type of latrine**	
Latrine with water (vs no latrine)	−1.26 (−2.39, -0.13)
Latrine without water (vs Adobe)	0.69 (0.19, 1.20)
**Type of household roof**	
Straw (vs Roofing tiles/Metal sheet)	0.15 (−0.37, 0.61)
Canvas (vs Roofing tiles/Metal sheet)	−0.58 (−1.50, -0.18)
Temperature*	−0.29 (−1.18,0.52)
Rainfall*	0.45 (−0.50,1.57)
Distance to lagoons*	0.74 (−0.54,1.96)
Distance to health care centre*	0.75 (0.06,1.69)
Distance to rivers*	−0.98 (−1.92,-0.07)
**Intercept**	−1.84 (−3.30,0.70)
**Rate of decay of spatial autocorrelation (*φ*)**	12.68 (2.97,19.70)
**Variance of spatial random effect**	4.61 (1.25,14.72)

*Variables were standardized to have mean = 0 and standard deviation = 1.

**Figure 4:**
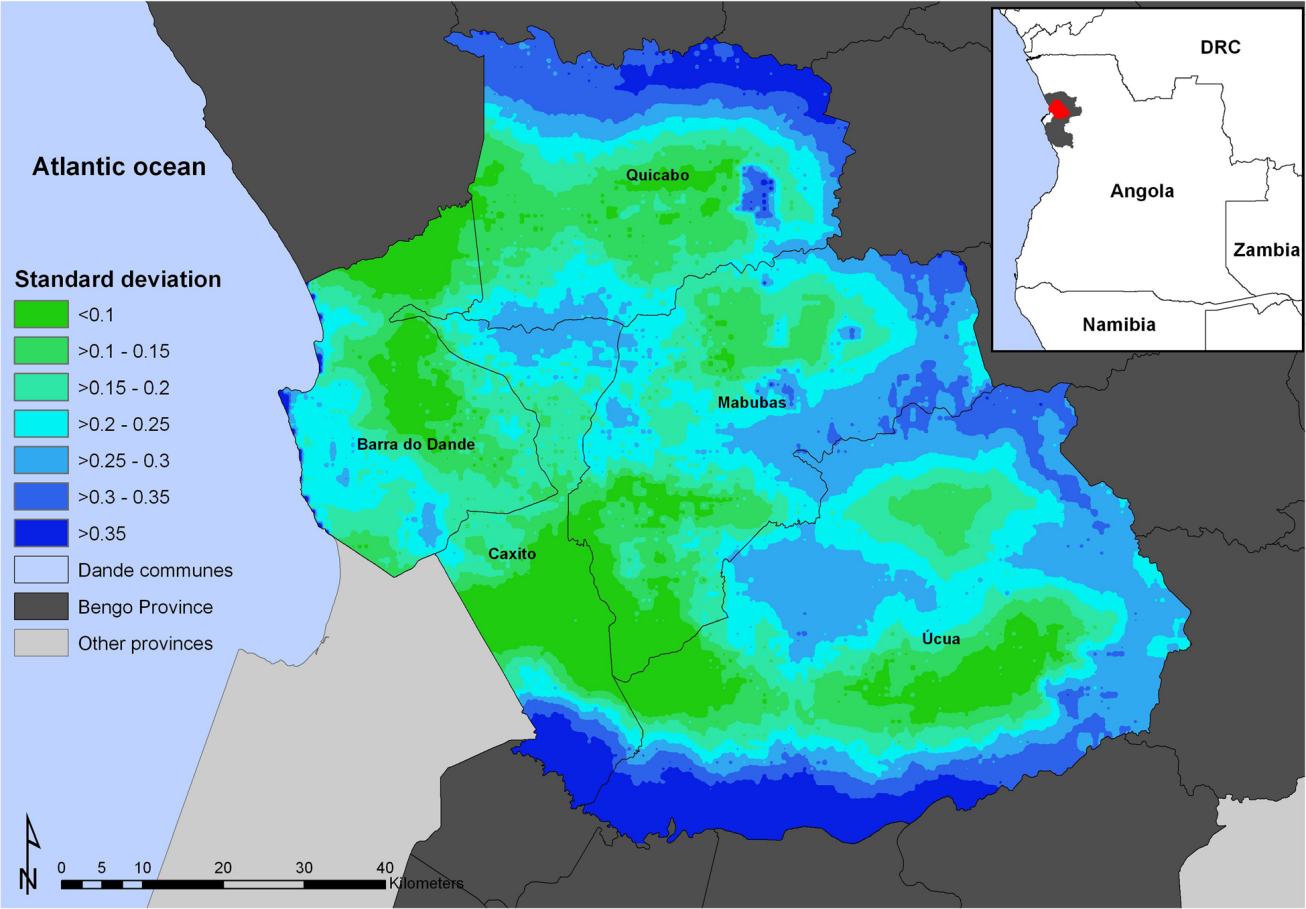
Standard deviation of predictive malaria in boys of older ages for the Dande municipality in Angola.

More circumscribed areas of high risk of malaria were found between the communes of Quicabo and Mabubas and between the communes of Mabubas and Úcua in central Dande. There is also a large elongated high risk area predicted to the south of the municipality in close proximity to inland water bodies. The map of predicted standard deviation (Figure 4) shows moderate prediction uncertainty (0.20<SD<0.35) for the large high risk region most of the eastern areas of the Dande municipality, and low to moderate prediction uncertainty (0.10<SD<0.20) for the circumscribed areas of high risk of malaria between the communes of Quicabo and Mabubas and between the communes of Mabubas and Úcua in central Dande; the highest prediction uncertainty (SD>0.35) was found in the large high risk region covering the north of the Dande municipality and in the large elongated high risk area predicted to the south of the municipality. The final predictive model showed acceptable predictive ability (i.e. AUC >70%).

### Comparison with previous larger-scale studies in the region

While the 2–10 year old global malaria map 2011 predictions for the Dande municipality was 41% (range: 33-47%) (see Additional file 1) and the national malaria map predictions for children aged <5 years the Bengo province was 24% (model-based prevalence adjusted for population), this study found predicted an overall prevalence (children aged <15 years) of 25% (range: 0.1-90%). Visually the predictive malaria map (Figure 2) shows considerable spatial heterogeneity compared to the global malaria map 2011 (see Additional file 1) and the national malaria map [26] predictions for the Dande municipality.

### Estimation of at-risk populations for malaria

The results show that in the Dande municipality 4,908 children aged <15 years were predicted to be at-risk of malaria in 2011 (Table 3). Most children predicted to be at risk were in the commune of Caxito (53.9%), followed by Quicabo (17.6%), Mabubas (16.5%), Úcua (8.4%) and Barra do Dande (3.6%).

**Table 3:** Predicted malaria prevalence in children aged ≤15 years by commune and estimated population at-risk (≤15 y.o.) in 2011 for the Dande municipality, Angola

**District**	**Mean predicted prevalence (%)**	**Target population at risk for 2011**
**Caxito**	17.2%	3,191*
**Barra do Dande**	10.5%	214†
**Quicabo**	26.1%	1,040†
**Mabubas**	26.9%	976 *
**Úcua**	31.6%	495*
**Total**	22.5%	5,916

^*^Assumes the demographic census of the CISA DSS, conducted in December 2011.

†Assumes a population growth rate 2005–2011 of 2.70% and proportion of <15 yo of 28.5% for 2011. Source: Population Division of the Department of Economic and Social Affairs of the United Nations Secretariat, *World Population Prospects: The 2010 Revision*, http://esa.un.org/unpd/wpp/index.htm.

## Discussion

This work shows the first detailed malaria risk map for children ≤15 years for an area of Angola for which no previous reliable information was available. The distribution and level of malaria endemicity estimated in the analysis reveals significant spatial variation in malaria risk, which previous mapping studies failed to convey. The study also identified important individual and modifiable household factors significantly associated with malaria risk, some of which with important operational relevance to the implementation of current malaria control strategies in the area.

### Drivers of spatial variation in malaria risk

This study, confirms the role of maternal malaria awareness at explaining the spatial variation in *Plasmodium* infection in children aged ≤15 years in the region [6]. Previous research in the study area had shown that, close to half of all children (48.8% of preschool and 45.5% of school-aged children) had received treatment for malaria in the past (therapeutic regimen unknown) [6]. In this report, previous malaria treatment was positively associated, although not significantly at the 5% level, with malaria risk in children. A likely explanation for this finding is that people are more likely to seek treatment in malarious areas. Alternatively this may also suggest that resistance to anti-malarial drugs, may be a concern in the study area [33]. Even though artemisinin based combination therapy is in place as first line therapy of confirmed malaria cases, it is unknown whether monotherapies are still being used by the population. Furthermore, recent epidemiological studies have shown the presence of sulphadoxine/pyrimethamine (SP) resistance markers in Angola [33-35]. Access to treatment is also an important indicator of the success of the malaria control in that interventions need to reach remote communities at increased risk of infection. With that regard, the study has shown that communities at greater distance from health care centres were at increased risk of malaria, suggesting that remoteness is an important risk factor for *Plasmodium* infection in Dande municipality.

Proximity to breeding sites has been shown to increase the likelihood of exposure opportunities to mosquitoes and with that regard, the results confirm that households closer to rivers are at increased risk of *Plasmodium* infection [36,37]. While the positive association between malaria and distance to lagoons was surprising and deserves further investigation, it can partly be explained by the sparseness of the data and the fact that very few individuals live in close proximity to lagoons in the study area. Household characteristics have also been shown to increase the likelihood of exposure opportunities to mosquitoes; for example, some studies have suggested an increased risk of malaria infection in houses made with vegetable material, which provides favourable conditions for mosquito survival [38-40]. The results show that the risk of *Plasmodium* infection was increased in households with straw roofing compared to those living in houses with tile or metal sheet roofing, but this was not statistically significant. Surprisingly, the results also show that households with canvas (Portuguese word “*lona*”) roofing were at a significantly reduced risk of malaria infection compared to households with tile or metal sheet roofing, and that households which have latrines without water were at increased risk of malaria. These findings warrant further investigation. The loss of statistical support for variables of the physical environment, such as rainfall and temperature is not surprising at this spatial scale and by the relative small variation in altitude.

### Identifying small-scale spatial variation in malaria

The significant unexplained variation in malaria risk related to survey location (as assessed by the residual semivariogram) suggested that considerable malaria clustering was left uncounted by variables included in the non-spatial multivariable model. This finding justified the need of a formal modelling of the second-order spatial variation using a MGB modelling framework.

Using a MBG model of malaria prevalence, the results show that malaria risk increases from west to east in the Dande municipality in line with previous work [10,26]. However, in contrast to previous spatial modelling work in the area the map developed in this study shows significant small-scale spatial variation in malaria endemicity. Compared to a global malaria model, lower malaria endemicity was predicted in the area (i.e. overall prevalence 25% compared to 41%) [10]. Compared to a national map [26], the results show that in the Dande municipality there is a greater variation in infection prevalence, from areas with prevalence < 10% to areas with infection prevalence > 60%. The map by Gosoniu *et al.*[26] shows a predicted parasitaemia prevalence range between 15 and 30%. Also the maps generated by the global- and national-level models fail to capture the important hot-spots of malaria (i.e. high risk areas of prevalence>50%) in the inland communes of Quicabo, Mabubas and Úcua.

The discrepancies between the mapped outputs from this study and those of global and national models (with respect to the small-scale spatial heterogeneity in malaria risk and the malaria endemicity estimates) may be linked to issues related to data sparseness and to the inherent limitations of the methodological approaches used in spatial modelling. In the case of the global model, it included historical malariometric data for a wide range of age groups, which by the time of modelling, consisted of 22,212 quality-checked and spatiotemporally unique data points – however, only two data points were available from our study area [10]. While the national model included 92 data points from the 2007 malaria indicator data only one data point was from the study area [26]. This suggests that global and national models are unable to provide an accurate estimation of malaria endemicity at small spatial scales, particularly in areas for which up-to-date empirical data are not available. In addition, the methodological approaches were different in both global and national models. While the global model used a spatiotemporal MBG formulation and predicted the *Pf*PR for the year 2010 age-standardized to the two to 10 year age-range [10], the national model used a purely spatial MBG model and generated a predictive map for the children aged <5 years [26]. Another methodological reason for the disparity may be the absence of individual- and household-level factors in the global and national models which are known to be better predictors of malaria endemicity at small spatial scales [41]. Neither of the global and national models included this information and for that reason these may fail to capture the small-scale spatial variation in exposure opportunities which are more likely to be driving malaria risk at small spatial scales.

Targeting malaria interventions to hotspots in areas of low malaria endemicity can help achieve elimination and with that regard maps can play a crucial role [7]. This is the first study showing that sub-national malaria mapping outperforms global and national malaria spatial risk models at identifying hotspots of malaria within a seemingly meso-endemic area. These results demonstrate that there may be a need to generate higher resolution malaria risk maps if maps are to be useful spatial decision support tools to inform local decisions on malaria control and the delivery of targeted malaria interventions. An added benefit of a higher resolution approach to malaria spatial risk modelling is that it allows the collection of individual- and household-level information. The inclusion of detailed information about health seeking behaviour and treatment availability and access to inadequate water, sanitation and housing allows the identification of modifiable risk factors (such as the ones identified in this study) which have the potential to be acted upon. However, the advantages of designing and conducting sub-national malaria mapping have to be weighed alongside budgetary constraints for their implementation. Nevertheless, this work provides important new knowledge regarding the existence of malaria hot-spots in the area that continental and national models failed to convey and thus can constitute important cartographic resources to target malaria control in the Dande municipality.

### Implications to malaria control in the CISA DSS area

The results shown that household construction materials, particularly roofing materials, are associated with malaria risk in the study area. Previous studies have shown that household modifications and improvements have been associated with a reduction of anopheline mosquitoes entering the house and a decrease of malaria infection prevalence and anaemia [42-44]. While currently in the CISA DSS area there are no IRS activities, the results suggests that if IRS is planned for the region, teams should be trained to spray not only the interior walls of houses, but the ceilings and underside of roofs as well, particularly where vegetation is used for roof construction. When financially possible, household modifications and improvements should also be considered. Importantly, the results confirm the likely successful impact that public health awareness campaigns targeting mothers and improving access to treatment can have at reducing *Plasmodium* infection risk in children in the region.

The study found considerable small-scale spatial variation in malaria risk indicating that there is scope for the predictive map to help inform spatially targeted interventions, such as the delivery of LLINs, IRS and IPT, to high risk groups (i.e. young children and pregnant women) in the CISA DSS. Areas of priority include important hot-spots of malaria (i.e. high risk areas of prevalence>50%) in the inland communes of Quicabo, Mabubas and Úcua. The low to moderate prediction uncertainty estimated to these areas suggest that targeting interventions to these areas is likely to contribute to a more efficient reduction of severe morbidity and mortality as well as to reduce malaria transmission. The approach conducted in this study was also important for estimating the burden of malaria in the Dande municipality in that that approximately six thousand children aged ≤15 years were predicted to be at risk of malaria in the region, half of them in the commune of Caxito.

The findings reported in this study need to be interpreted in light of study limitations: firstly, it is possible that the lower infection prevalence levels confirmed by this study compared to the continental model can be due to the timing of the field-work between May and beginning of August (the end of the rainy season and beginning of the dry season). Nevertheless, the overall endemicity found in this study is in line with the results for the Bengo province from the 2011 MIS survey [4]. Secondly, the sparseness of the data used in the analysis may limit the ability to detect associations (statistical power) by not identifying sufficient small-scale spatial heterogeneity. This is particularly noticeable in inland areas where prediction uncertainty was estimated to be highest. Finally, recall bias may be present for mothers responses to questions related to malaria treatment and the questions of previous treatment did not consider when treatment was administrated and what treatment regimen had been given.

## Conclusions

The distribution and level of malaria endemicity estimated in this analysis reveal significant spatial variation in malaria risk which previous mapping studies failed to convey. This suggests that there may be a need to generate higher resolution maps in seemingly mesoendemic areas. This study identified important individual and modifiable household factors significantly associated with malaria risk. The findings have important operational relevance to the implementation of the current malaria control strategies in the area.

## Competing interests

The authors declare that they have no competing interests.

## Authors’ contributions

RJSM: prepared the dataset, performed data analysis and wrote the manuscript; SVN: designed, carried out the survey, prepared the dataset and contributed to the manuscript; AL: prepared the dataset and contributed to the manuscript; JCSF: contributed to study design and implementation and revised the manuscript; ACAC: helped design the survey and contributed to the manuscript. All authors read and approved the final manuscript.

## Supplementary Material

Additional file 1 The data provided represent a description of the statistical notation used in model-based Bayesian geostatistical prediction and map of malaria endemicity for the study region derived from the global map of malaria. Click here for file
