# Supplementary material for: Finding malaria hot-spots in northern Angola: the role of individual, household and environmental factors within a meso-endemic area
Source: Malar J. 2012 Nov 22;11:385. doi: 10.1186/1475-2875-11-385 (PMC3519509; doi:10.1186/1475-2875-11-385)
Supplement: Additional file 1 — The data provided represent a description of the statistical notation used in model-based Bayesian geostatistical prediction and map of malaria endemicity for the study region derived from the global map of malaria. [file 1475-2875-11-385-S1.doc]

**Finding malaria hot-spots in Angola: the role of individual, household and environmental factors within a meso-endemic area.**

**Technical appendix**

***Model-based Bayesian geostatistical prediction***

For the purpose of Bayesian geostatistical modelling the individual malaria infection status is considered a binary outcome variable *Y_i_* which was labelled *Y_i_* =1 for malaria infected individuals and 0 for non-infected individuals. The models used assume a conditional Bernoulli model for the binary outcome variable where the probability *p* of an individual *i* being infected, given the location *j* of the individual is given by:

$$Y{}_{i,j}\sim Bernoulli(p_{i,j})$$

$$logit(p_{i,j})=\alpha+\sum_{k=1}^{p} \beta_{k}\times x_{i,j}+u_{i}$$

where *Y_i,j_* is the infectious status of an individual in location *j*, *p_i,j_* is the probability of an individual being a case in location j, *α* is the intercept, *x_i,j_* is a matrix of covariates, *β* is a vector of coefficients and *u_i_* is a geostatistical random effect defined by an isotropic exponential spatial correlation function:

$f(d_{ab};\varphi)=\exp\left[-(\varphi d_{ab})\right]$,

where *d_ab_* are the distances between pairs of points *a* and *b*, and $\varphi$ is the rate of decline of spatial correlation per unit of distance. Non-informative priors were used for *α* (uniform prior with bounds -$\infty$ and $\infty$) and the coefficients (normal prior with mean = 0 and precision = 1 × 10^-4^). The prior distribution of $\varphi$ was minimum 1 and maximum 600 [i.e. phi ~ dunif(1, 600)]. The precision of *u_i_* was given a non-informative gamma distribution [i.e. tau ~ dgamma(1,0.05)].

The prediction of the prevalence of infection was performed by *kriging* the geostatistical random effect and adding it to the sum of the products of the coefficients for the fixed effects and the values of the fixed effects at each prediction location. This was done using the *spatial.unipred* kriging function. This function implements independent simulations that do not consider neighboring values, as opposed to joint prediction which is conditional on the values of neighboring locations. While joint prediction yields more accurate measures of prediction uncertainty, it was not considered feasible in this study due to having extremely demanding computational requirements.The overall sum was then back-transformed from the logit scale to the prevalence scale, giving prediction surfaces for prevalence of infection.

A burn-in of 5,000 iterations was allowed, followed by 10,000 iterations where values for the intercept, coefficients and predicted probability of infection at the prediction locations were stored. Diagnostic tests for convergence of the stored variables were undertaken, including visual examination of history and density plots; convergence was successfully achieved after 5,000 iterations. The outputs of Bayesian models including parameter estimates and spatial prediction are termed posterior distributions. These distributions fully represent uncertainties associated with estimated values. We summarized the posterior distributions in terms of the posterior mean and 95% Bayesian credible interval (CrI), within which the true value occurs with a probability of 95%.

***Global malaria map for 2010***

**
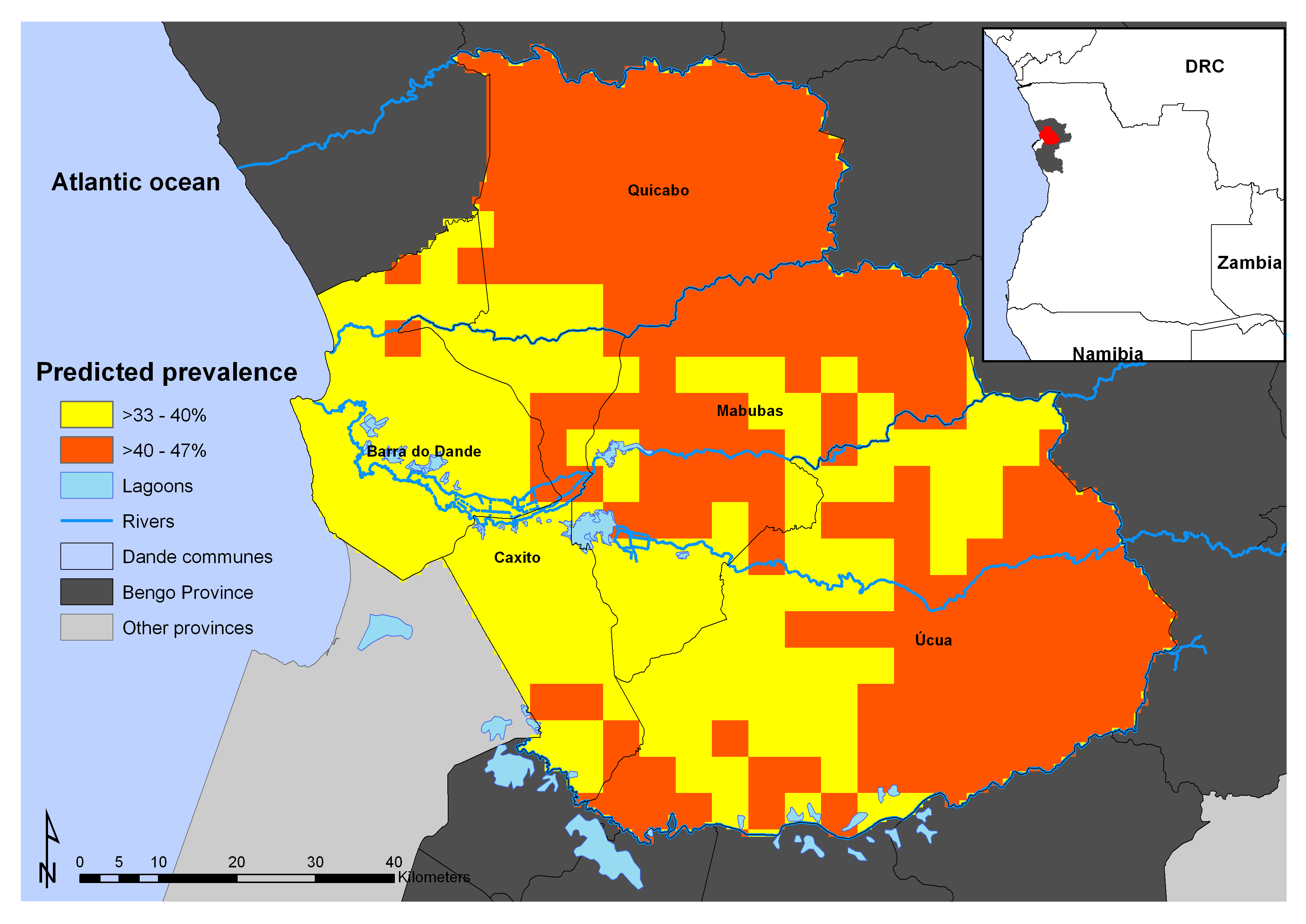
**

Figure S1 Mean predictive *Pf*PR_2-10_  for 2010 in Dande municipality, Angola, using a global malaria model.

**References**

1. Gething PW, Patil AP, Smith DL, Guerra CA, Elyazar IR, Johnston GL, Tatem AJ, Hay SI: **A new world malaria map: Plasmodium falciparum endemicity in 2010.** *Malar J* 2011, **10:**378.
